# Perception of health professions students of their role model status in Toombak cessation: A cross-sectional study from Sudan

**DOI:** 10.1371/journal.pone.0210837

**Published:** 2019-02-07

**Authors:** Hatim Mohammed Almahdi, Raouf Wahab Ali, Anne Nordrehaug Åstrøm, Elwalid Fadul Nasir

**Affiliations:** 1 University of Science and Technology, Faculty of Dentistry, Omdurman, Sudan; 2 King Faisal University, College of Dentistry, AlAhsa, Saudi Arabia; 3 Centre for International Health, University of Bergen, Bergen, Norway; 4 Department of Clinical Dentistry, Faculty of Medicine and Odontology, University of Bergen, Bergen, Norway; Tribhuvan University Institute of Medicine, NEPAL

## Abstract

**Introduction:**

Health professionals are to play an essential and effective role in tobacco control. Toombak is a popular form of smokeless tobacco, locally made and used among Sudanese. It is associated with oral and systemic health hazards, particularly oral cancer. This study was set out to assess health professions students’ perception of their status as future role models for their patients and the public regarding Toombak use cessation and to explore socio-cognitive covariates of perceived role model status.

**Materials and methods:**

A cross-sectional study was conducted in 2013–2014 using a self-administered questionnaire among health professions students enrolled in Medicine, Pharmacy, Dentistry and Nursing colleges in Khartoum state, Sudan. A total of 1530 participants were recruited from colleges using a cluster stratified sampling procedure.

**Results:**

The majority of the participants (88.2%) were 19–22 year old, females showed predominance (66.1%). Most of the participants (69.7%) scored good knowledge of Toombak-related therapies. The majority (97%) had a positive attitude towards banning Toombak use in public. Half of the participants (50.4%) confirmed availability of Toombak control policy in their college, while 66% reported receiving training in Toombak cessation. Only 9.2% of the participants reported that they have ever used Toombak; among them, 69.5% were current daily users. Less than half (47.8%) reported a positive perception of their status as a role model. Logistic regression model revealed that tobacco cessation training, availability of college policy and positive attitudes towards banning Toombak use in public were strong predictors of with a positive perception of role model status.

**Conclusion:**

Imposing Toombak control policies within colleges combined with formal training in cessation methods, would reinforce the positive perception of health professions students as a role model, particularly the students themselves seem to be ready for such systematic training.

## Introduction

The tobacco epidemic is one of the main public health problems the world has ever met [1]. It is estimated that more than 300 million adults in 70 different countries use a form of smokeless tobacco (SLT); the overwhelming majority (250 million) are in low and middle-income countries (LMIC) [2]. The burden of diseases due to use of SLT is estimated to be more than six million DALYs (*Disability- Adjusted Life Years*) and over a quarter of a million deaths occur each year [3]. Cessation of tobacco use reduces its harmful effects, namely; blood pressure, the risk of coronary heart diseases and stroke, improves lung function and increases life expectancy [4].

Toombak is a form of SLT locally made and consumed in Sudan. It is used as an oral snuff and is made from finely grounded leaves of *Nicotiana rustica*. Toombak has the highest level of nicotine and carcinogens such as tobacco-specific nitrosamines (TSNAs) and nicotine-derived nitrosamine ketone (NNK) levels which are 50–100 times greater than the maximum concentration found among other tobacco products in other parts of the world [5, 6]. It is etiologically related to oral diseases such as dental caries, periodontal disease, and may eventually result in tooth loss [7–9]. In Sudan, several epidemiologic studies have shown a significant association between Toombak use and oral cancer [10–12]

World Health Organization (WHO) defined health professionals to include “physicians, nurses, midwives, dentists, psychologists, psychiatrists, pharmacists, chiropractors and other health-related professionals” [13]. Health professionals can act as a role model for their patients in prevention and tobacco cessation and are in a position to help their patients by giving advice and guidance [14, 15].

Health professionals’ role model status is determined by the fact that they are recognised to be health experts and the most knowledgeable than patients and society [13]. They are expected to act on the basis of this knowledge and furthermore, to advocate for the health of their patients and community [16]. It is confirmed that the chances of tobacco cessation increase after counselling by health professionals [17].

Perceptions of health professionals of their status as a role model for their patients in tobacco cessation and prevention may be reflected by their attitudes towards tobacco use, and by their advice to patients and the public about tobacco cessation and prevention [18, 19]. This perception of role model status is strongly influenced by the specialized training health professionals have received during the study period [20]. Evidence suggests that formal training on tobacco cessation play an important role to shape the attitudes of health professions students (HPS) towards tobacco cessation and improve their contribution to tobacco control [21].

WHO support the inclusion of tobacco cessation programs in health-education and services, and that all health providers should become advocates for tobacco control [22]. Worldwide, few HPS reported having received formal training in tobacco cessation, while the majority believed that such training is required [15, 23–26]. In Sudan, HPS who reported having received formal training about Toombak cessation in their curricula ranged from 5.8% (Pharmacy students), 31.6% (Medical students, 28.1% (Dental students), to 53.6% (Nursing students) [27].

Whereas previous research has explored HPS’ attitudes towards and use of tobacco, few studies have considered the use of Toombak and HPS perception of themselves as role models for their patients and public in Toombak cessation activities. Thus, more information is needed to focus on the use of Toombak, as it is used by almost 38% of the Sudanese people [28]. This study aimed to assess the perception of the HPS and their status as future role models for the patients and the public regarding Toombak use cessation and to explore socio-cognitive covariates of the perceived role model status.

## Materials and methods

A cross-sectional study was conducted using a self-administered questionnaire among HPS enrolled in Medicine, Pharmacy, Dentistry and Nursing colleges in Khartoum state, Sudan, during 2013–2014.

### Sampling procedures

In Khartoum state, a total of about 20,000 health professions students were enrolled in 55 colleges during the study period. There are 18 colleges offering medical degree, 15 offering pharmacy degree, 13 offering dental degree, and nine offering nursing degree [29]. Using cluster stratified sampling procedures, the colleges (clusters) were stratified by discipline (Medicine, Pharmacy, Dentistry and Nursing) and each discipline stratified by type (public/private) and then by gender (male/female) proportion to the size of the stratum. Using simple random procedure eight colleges were selected. It has been estimated that the eight colleges (one public and one private for each discipline), would provide the sample size that had been calculated beforehand which amounts to 1442 students consisting of; 735 Medicine students, 375 Pharmacy students, 173 Dentistry students and 159 Nursing students. Third-year students in the selected colleges were invited to participate, a total of 1530 students were recruited. Eligibility criteria required was the presence of the third-year students at the time of the study and having signed an informed consent form.

### Data collection

The questionnaire and study information sheet were administered in English as it is the language used for teaching among the HPS in Sudan. A brief introduction to the research objectives was provided by the researcher. No personal identifying information was obtained and therefore the individual responses were kept anonymous.

The questionnaire used in this study was based on the Global Health Professional Students Survey (GHPSS) questionnaire. The GHPSS is a college-based survey of 3rd-year students pursuing degrees in Medicine, Pharmacy, Dentistry, Nursing and other health professions, that has been developed by the WHO, the Centres for Disease Control and Prevention (CDC, USA) in 2005 [30]. The final form of the questionnaire is divided into five areas: *Demographic characteristics* (3 items), *knowledge of Toombak-related therapies* (2 items), *Toombak* cessation *training information and college policy* (6 items), perceived *role of health professions students* (4 items) *and attitudes toward the Toombak control* (3 items), and *Toombak use* (2 items). A pilot study was carried out among 150 HPS (included in the study). Accordingly, no further major changes were made to the questionnaire.

### Questions and variables

#### Demographic characteristics includes gender, age group, type of study

*Knowledge of Toombak cessation therapies* was measured by *two* questions “have you ever heard of using nicotine replacement therapies in tobacco”; “have you ever heard of using antidepressants in tobacco cessation programs”; using response options (1) “yes”; (2) “no”; (3) “I don’t know”. The original categories were recoded into (0) “no” (including original responses 2, 3); (1) “yes” (including original responses 1). A sum variable labelled “*knowledge of Toombak cessation therapy*” (r (0.38), *p* <0.01) was constructed from the two questions (0–2). This sum was recoded into (0) no knowledge (including original categories 0); (1) good knowledge (including original categories 1, 2).

*Toombak cessation training at college* was measured by *five* questions; “during your college training, have you ever received any formal training in Toombak cessation approaches to use with patients”; “did you taught in any of your classes about the health hazards of Toombak”; “did you discuss in any of your classes the reasons why people use Toombak”; “did you learn that it is important to record Toombak use history as part of a patient’s general medical history”; “did you learn that it is important to provide educational materials to support Toombak cessation to patients who want to quit Toombak”. Using response options (1) “yes”; (2) “no”; (3) “I don’t know”; the original categories were recoded into (0) no training (including original categories 2, 3); (1) yes, there is training (including original categories 1). A sum variable labelled “*Toombak cessation training at college*” (Cronbach’s α = 0.94) was constructed from the five questions (0–5) and recoded into (0) no training (includes 0); (1) yes, there is training (including original categories 1, 2, 3, 4, 5).

One question measured *availability of college policy;* “does your school have an official policy banning Toombak use in school buildings and clinics”; using response options (1) “yes, for school buildings only”; (2) “yes, for clinics only”; (3) “yes, for both school buildings and clinics”; (4) “no official policy”; the original categories were recoded into (0) no (including initial response 4); (1) yes (including initial responses 1, 2, 3).

Three questions measured *attitude towards banning Toombak use in public*; “should Toombak sales to adolescents (persons younger than 18 years old) be banned”; “should Toombak be banned in restaurants” and “should Toombak be banned in parties”; using response options (1) “yes”; (2) “no”; (3) “I don’t know”; the original categories were recoded into (0) “negative attitude” (including initial responses 2, 3); (1) “positive attitude” (including initial response 1). A sum variable “*attitude towards banning Toombak use in public*” (Cronbach’s α = 0.88) was constructed from the three question (0–3) and recoded into (0) positive attitude (including original categories 0); (1) negative attitude (including original categories 1, 2, 3).

*Ever users* of Toombak mean having used Toombak at least once or twice in their life. *Current users* of Toombak were defined as having used Toombak at least once in the last 30 days either daily or near-daily [31].

*Ever use of Toombak* was measured by *one* question; “have you ever tried or experimented with Toombak, even one or two snuffs” using response options (1) “yes”; (2) “no”.

*Health professions students’ perception of their status as future role models in Toombak cessation* was measured by *four* questions; “do health professionals serve as “role models” for their patients and the public”; “should health professionals routinely advise their patients to quit Toombak”; “do health professionals have a role in giving advice or information about Toombak cessation to patients”; “are patients’ chances of quitting Toombak increase if a health professional advises him or her to quit”. Using response options (1) “yes”; (2) “no”; (3) “I don’t know”; the original categories were recoded into (0) negative perception (including original responses 2, 3); (1) positive perception (including original response 1). A sum variable “*perceived role model*” (Cronbach’s α = 0.90) was constructed from the four question (0–4) and recoded into (0) negative perception (including original categories 0, 1, 2, 3); (1) positive perception (including original categories 4) [32].

### Statistical analyses

Data were analysed using the Statistical Package for the Social Science, version 20 (IBM SPSS Statistics). Descriptive analyses were performed using frequencies and percentages. Cross-tabulation using Chi-square, Odds Ratio (OR), and Confidence Interval (CI) assessed bivariate relationships between the dependent variable (perceived role model) and all independent variables. Multiple variable analysis was conducted using logistic regression with those independent variables that showed significant associations with the outcome variable in the bivariate analysis. Estimates were presented as OR and 95% CI, in addition to Nagelkerke’s (R^2^) to the level of explanation in the variance of the model.

#### Ethical consideration

Ethical approval was obtained from Directorate of Research, Ministry of Health, Khartoum state. The Directorate reference was No. WKH/WS/AA/AB.18.09.2013. Written informed consent was attained from the participating students and the participation was voluntary and anonymous.

## Results and discussion

A total of 1530 HPS participated in the present study. Among the participants, 41.3% were in Medicine, followed by Pharmacy (29.7%), Nursing (14.8%) and Dentistry (14.2%). The majority of the students (88.2%) were 19–22 years. The gender distribution showed a predominance of females (66.1%), and this was the trend in all subgroups except for medical students (Table 1).

**Table 1:** Sample profile: Socio-demographic distribution % (n) of participants in each health professional sub-group.

Characteristics	Medicine	Pharmacy	Dental	Nursing	Total
Subgroup	41.3% (632)	29.7% (455)	14.2% (217)	14.8% (226)	100% (1530)
**Age group**					
(15–18) years	20.6% (122)	4.4% (18)	11.6% (24)	1% (2)	11.8% (166)
(19–22) years	79.4% (470)	95.6% (394)	88.4% (183)	99% (197)	88.2% (1224)
**Gender**					
Female	48.6% (302)	25.2% (114)	77.8% (168)	85.3% (192)	66.1% (1001)
Male	51.4% (319)	74.8% (455)	22.2% (48)	14.7% (33)	33.9% (514)

Most of the participants (69.7%) scored good knowledge of Toombak-related therapies. Almost all of the participants (97%) reported positive attitude towards banning Toombak use in public. Half of the participants (50.4%) confirmed availability of Toombak control policy in their college, while 66% reported receiving training in Toombak cessation.

Only 9.2% of the participants reported that they have ever used Toombak; among them, 69.5% were current daily Toombak users. The highest frequency of Toombak use was among Medicine students (53.6%) and the lowest was among Nursing students (4.3%).

**Fig 1:**
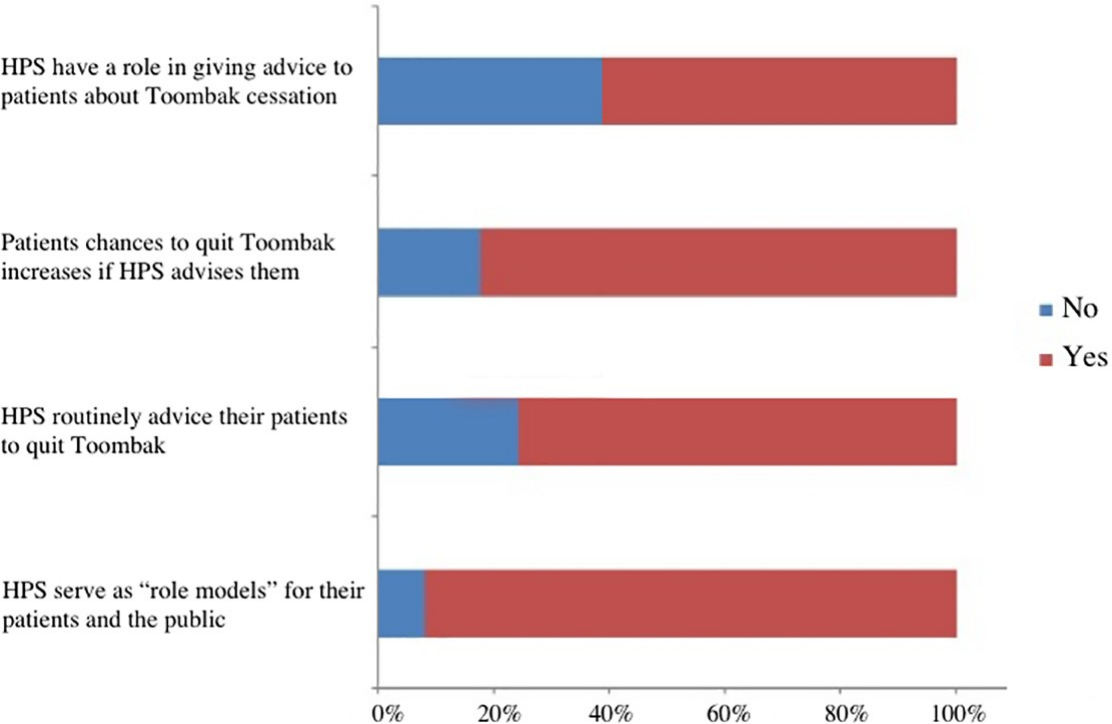
Frequency and percentages % (n) of students’ perceived status as a role model in Toombak cessation by health professions students.

Almost half of the participants (47.8%) reported a positive perception of their status as a role model for their patients and the public regarding Toombak cessation. Among Nursing and Medicine students, Pharmacy and Dentistry students were 52.9%, 53%, 40.3% and 42.9%, respectively. Furthermore, most of the students (82.4%) believed that there is an increase in the chances of quitting tobacco if a health professional advises his/her patients to quit. The majority of the participants (75.6%) perceived that health professionals have a role in giving advice about Toombak cessation to their patients (Fig 1).

As shown in Table 2, a statistically significant difference in reporting a positive perception of their role model status among students confirming positive attitude towards banning Toombak use in public (48.6% versus 18.6%, p< 0.001), training on Toombak cessation (51.5% versus 43.4%, p≤0.001), and availability of college policy (51.2% versus 44%, p≤0.001) compared to their counterparts.

**Table 2:** Percentages, OR and CI of perceived positive role model status by demographic characteristics, formal training, college policy, Toombak use, knowledge and attitudes towards Toombak cessation.

Characteristics	Positively perceived status as role model % (n)	OR (CI)
**Age group**		
(15–18) years	41.2 (66)	1
(19–22) years	48.4 (585)	1.33 (0.95–1.86)
**Gender**		
Female	47.3 (462)	1
Male	48.1 (238)	11.03 (0.83–1.27)
**Knowledge about Toombak cessation therapy**		
Poor knowledge	47.3 (207)	1
Good knowledge	48 (489)	1.03 (0.82–1.29)
**Attitude towards Toombak use in public**		
Negative attitude	18.6 (18)	1
Positive attitude	48.6 (691)**	4.13 (1.90–8.96)
**Training on Tobacco cessation**		
No	43.4 (272)	1
Yes	51.5 (416)**	1.38 (1.12–1.71)
**Availability of college policy**		
No	44 (305)	1
Yes	51.2 (362)**	1.33 (1.08–1.64)
**Toombak use**		
Non- users	48 (648)	1
Ever user	45.9 (61)	0.91 (0.64–1.31)

*p≤0.05

**p≤0.001

Perceived status as a role model was regressed on age, gender, attitude towards banning Toombak use in public, training on tobacco cessation and availability of college policy. This model revealed a Nagelkerke’s R^2^ of 0.022, explaining 2.2% of the variance perceived role model status. The final model indicated that tobacco cessation training, availability of college policy and attitudes towards banning Toombak use in public were significantly associated with a positive perception of role model status. As shown in Table 3, students who did confirm training on tobacco cessation were more likely to have a positive perception of role model status as compared to their counterparts. Students with reported college policy had a higher likelihood reporting positive perception of their role model compared to those with no college policy. Finally, students who had a positive attitude towards banning Toombak use in public were more likely to perceive a positive role model status (OR 1.26, CI 1.00–1.59), (OR 1.25, CI 0.99–1.58) and (OR 3.45, CI 1.38–8.61), respectively (Table 3).

**Table 3:** Health professions students’ perceived role model status regressed on formal training, availability of college policy, and attitude towards use of Toombak in public (OR, CI).

Characteristics	Role model OR (95% CI)
**Age group**	
(15–18) years	1
(19–22) years	1.27 (0.89–1.82)
**Gender**	
Female	1
Male	1.06 (0.82–1.36)
**Training on Tobacco cessation**	
No	1
Yes	1.26 (1.00–1.59)*
**Availability of college policy**	
No	1
Yes	1.25 (0.99–1.58)*
**Attitude towards Toombak use in public**	
Negative attitude	1
Positive attitude	3.45(1.38–8.61)**

R^2^ = 0.022

*p≤0.05

**p≤0.001

## Discussion

This study was carried out to investigate Sudanese HPS’ perceived status as a role model in Toombak control and its possible covariates. It revealed that less than half of the participants reported a positive perception regarding HPS’ role model status in Toombak cessation. Moreover, perceived role model status was associated with their attitudes towards banning Toombak use in public, the availability of training and college policy about tobacco control.

The findings of this study showed a lower percentage of students confirmed a positive perception of their role model status in Toombak cessation than a recent study by Sreeramareddy [33], in which 70% of the students globally agreed that health professionals are role models in tobacco cessation to their patients and public. In addition, previous studies in Sudan showed that between 62–75% of HPS had a positive perception of being a role model to their patients and public in tobacco cessation [27]. This finding may predict the role these students might play in Toombak control in the future, as it highlights a critical behavioural conceptual aspect as the evidence available confirmed that the success of any tobacco cessation program depends on the involvement of the healthcare personnel and it is associated with their positive perception towards their role in tobacco cessation [34–36].

One of the important covariates of a positive perception of role model status in this study is the formal training included in the curriculum of HPS. A total of 44% of the HPS reported no formal training in tobacco cessation in the curriculum. This finding is in accordance with several studies confirming that the lack of enough formal training is a widespread problem [33, 37–39]. The inclusion of tobacco cessation in the curriculum of the medical schools in low-income countries is faced with multiple barriers, such as lack of available teaching time, staff and financial resources, limited knowledge of the teachers in this field, no plans and organizational problems such as inflexibility of medical curricula to introduce new topics [26, 40]. Tobacco training programs can improve knowledge of tobacco cessation advice giving as well as attitudes, behavioural change and skills among HPS [41, 42].

This study reported disparities among the HPS formal training of Toombak cessation, as a higher percentage of Nursing students reported to have formal training than their counterpart in other colleges. This is consistent with 2007 GHSPS in Sudan [27]. The disparity in formal training among different categories of HPS was reported in other studies e.*g*. In Greece, the pharmacy students were reported the least trained group [25]. Therefore, a multidisciplinary effort from all healthcare workers would to be the best target, Future dentists; pharmacists may, therefore, be ideally placed to work with other health workers in helping to reduce the burden of tobacco use among patients [43].

This study reporetd that students who did agree with having a positive attitude towards banning Toombak use in public places, were most likely to have a positive perception of their status as a role model. Studies have reported attitudes towards tobacco ban, associated with their knowledge of harmful effects of tobacco use, and their own status of using tobacco were important factors in shaping their perception as a role model [44]. In addition, this may be explained by health behavioural models, which argue that an individual’s beliefs may lead to certain attitudes and consequently to certain behaviours regarding the same health issue [45].

This study has various limitations; firstly, it focuses on third-year HPS, which has not had considerable contact with patients as the clinical education usually, starts during the third year of their professional education. Their perceptions might change over time. Secondly, the data are a self-reported, which can be subject to problems such as social desirability and recall bias. To cope with such potential bias, all measures to ensure the anonymity of the respondents and to keep the study anonymous was assured. The major strengths of this study are the use of a large, broadly representative sample of students from institutes in Khartoum, Sudan. The reliability of the questionnaire aligns with the GHPSS used before in Sudan. consistent evidence indicates that results obtained from such data are reliable [46]. In addition to high Cronbach’s α reliability test ranges (0.88–0.94).

The findings of this study may have direct implications for health education and the involvement of Sudanese health professions in the promotion of tobacco control. This study reports the influence of various factors on positive perceptions of HP students regarding their status as a role model for tobacco control in Sudan. Further research will be needed to confirm these findings, and determine whether these findings can be used to advocate the need to mould HPS perceptions.

## Conclusions

Imposed Toombak control policies within colleges combined with formal training in cessation methods, would reinforce the positive perception of HPS towards their status as role models to their patients and public towards Toombak control, particularly the students themselves seem to be ready for such systematic training. Thus, health colleges in Sudan should be encouraged to incorporate Toombak-related issues in curricula, and to design and implement training programs for HPS that will enable them to develop the basic skills and capacity to provide effective Toombak cessation methods to their future patients.

## Supporting information

S1 File (SAV)
